# Selective Use of Pulmonary Vasodilators in Patients with Fontan Physiology

**DOI:** 10.1155/2022/7602793

**Published:** 2022-11-10

**Authors:** Thomas Glenn, Nicole Duster, Jerry Dwek, Jose Silva-Sepulveda, Howaida G. El-Said

**Affiliations:** ^1^San Diego School of Medicine, Department of Pediatrics, Division of Pediatric Cardiology, University of California, San Diego, CA, USA; ^2^San Diego School of Medicine, Department of Pediatrics, University of California, San Diego, CA, USA; ^3^San Diego School of Medicine, Department of Radiology, Division of Pediatric Cardiology, University of California, San Diego, CA, USA

## Abstract

**Background:**

Fontan-associated liver disease is a well-known sequela following the Fontan procedure for patients living with single-ventricle heart disease. Pulmonary vasodilators, such as phosphodiesterase type 5 inhibitors, have emerged as a potential therapeutic option for lowering central venous pressures by reducing pulmonary vascular resistance.

**Method:**

We performed a single-center retrospective review of Fontan patients who were placed on pulmonary vasodilator therapy with prehemodynamic and posthemodynamic, MR elastography, and histologic assessments.

**Results:**

A total of 125 patients with Fontan circulation underwent surveillance with cardiac catheterization during the review period. Fifty-three (42%) patients who did not have increased end-diastolic pressures at the time of cardiac catheterization were started on phosphodiesterase type 5 inhibitor therapy. Nine patients (17%) underwent posttherapy follow-up catheterization. The mean Fontan pressure decreased from 15.4 ± 3.3 mmHg to 13.3 ± 2.5 mmHg (*p*=0.026), after initiation of pulmonary vasodilatory therapy. There was no change in end-diastolic pressure, transpulmonary gradient, wedge pressure, pulmonary vascular resistance, cardiac index, or saturation. Eleven patients (21%) underwent pretherapy MR elastography testing with posttherapy follow-up MR elastography. We found no improvement in liver stiffness score following the application of pulmonary vasodilators. Three patients underwent pretherapy and posttherapy liver biopsies, with variable histological changes observed within the hepatic parenchyma.

**Conclusions:**

These data demonstrate indeterminate results for the selective use of pulmonary vasodilators but highlight the need for large prospective randomized control trials of pulmonary vasodilator therapies to fully assess the benefit of such therapies in Fontan-associated liver disease.

## 1. Introduction

The Fontan procedure, which was first described in 1971, is generally accepted as the final staged palliation for patients with single ventricle physiology and has led to improved survival amongst this patient population [1, 2]. Despite this, Fontan and Baudet initially cautioned that “this procedure is not an anatomical correction, which would require the creation of a right ventricle, but a procedure of physiological pulmonary blood flow restoration, with suppression of right and left blood flow mixing” [1]. The absence of a subpulmonary ventricle and reliance on passive pulmonary blood flow directly lead to increased central venous pressure, and as such, this unfavorable physiologic state leads to chronic end-organ dysfunction, characterized by liver fibrosis, low bone density, renal disease, lymphatic dysfunction, lung disease, and cardiac dysfunction [3]. The liver, which is particularly susceptible to high venous pressures, also sees impaired arterial flow as a result of decreased cardiac output, as seen in this cohort of patients [4]. Fontan-associated liver disease is likely due to a combination of these factors, including both elevated central venous pressure and impaired arterial flow [4]. Hepatic abnormalities have been seen in patients even before the Fontan operation and as early as 20–35 days postoperatively following Fontan as a result of this physiologic change [5–7]. Hepatic fibrosis exists in all patients following Fontan operation, and its severity is associated with time since Fontan operation and elevated Fontan pressures [8, 9].

Throughout the last decade, many Fontan surveillance programs have evolved across the world, utilizing various invasive and noninvasive techniques to assess for Fontan-associated liver disease [3, 10–12]. As the full understanding of Fontan-associated liver disease continues to evolve, many management strategies have been developed aimed at optimizing this abnormal physiologic state. One such therapy, phosphodiesterase-5 inhibitors, acts on and promotes the cGMP-mediated cellular pathway, resulting in the creation of nitric oxide. Nitric oxide acts on receptors within the smooth muscle of the pulmonary vascular bed, causing relaxation of tissues, and thus, a drop in vascular resistance is achieved. Phosphodiesterase type 5 inhibitors have been shown to decrease pulmonary vascular resistance and improve cardiac output in patients with single-ventricle physiology [8, 13]. Other large studies, such as the Fontan Udenafil Exercise Longitudinal (FUEL) trial, have shown no associated improvement in oxygen consumption at peak exercise with patients treated with phosphodiesterase type 5 inhibitors. This study only demonstrated some improvement in markers of exercise performance at the ventilatory anaerobic threshold but did not evaluate the effect of udenafil on Fontan hemodynamics or the extent of Fontan-associated liver disease [14].

The purpose of this study is to describe the experience and outcomes of phosphodiesterase type 5 inhibitors on individuals living with Fontan physiology at Rady Children's Hospital, San Diego, and correlate the associated Fontan hemodynamic, liver magnetic resonance elastography, and hepatic histological findings.

## 2. Materials and Methods

This was a 6-year (2014–2020) retrospective review of all patients who underwent the Fontan operation and subsequently had a cardiac catheterization or MR elastography to evaluate for Fontan-associated liver disease at Rady Children's Hospital, San Diego. The practice at our institution is routine screening 5–10 years post-Fontan with cardiac catheterization, imaging of the liver by magnetic resonance elastography, and transjugular liver biopsy, when clinically indicated, or when Fontan pressure is greater than 15 mmHg or liver stiffness score by MR elastography is greater than 5 kPa [15]. Patients with a Fontan pressure greater than 15 mmHg and an end-diastolic pressure less than 10 mmHg, resulting in a transpulmonary gradient of at least 7 mmHg, were placed on a phosphodiesterase type 5 inhibitor following cardiac catheterization. All patients in our analysis who were placed on phosphodiesterase type 5 inhibitor therapy were started on tadalafil. Tadalafil was started at a low dose (0.5 mg/kg/day) and if tolerated, it was increased to a maximum dose of 1 mg/kg/day or 40 mg/day. The Institutional Review Board at Rady Children's Hospital San Diego approved the study. Data were collected by retrospective review of the medical records for patient demographics, cardiac diagnosis, medications, surgical procedures, hemodynamic data, MR elastography data, and histological data.

Only patients who were identified to have been started on phosphodiesterase type 5 inhibitor therapy and who underwent pretherapy and posttherapy cardiac catheterization or MR elastography were included in the analysis. Continuous data were summarized as the mean or median with a standard deviation or range when appropriate or as numbers with percentages for discrete data. A two sample *t*-test was used for continuous data as appropriate. A *p* value of less than 0.05 was considered statistically significant.

### 2.1. Standardized Approach to a Cardiac Catheterization

Cardiac catheterization was performed under general anesthesia with positive pressure ventilation. Hemodynamic measurements included standard pressure and saturation data under conditions of 21% FiO_2_. Typically, angiography was performed in addition to transcatheter interventions at the discretion of the primary operator based on catheterization findings. These data were not utilized in the present study.

### 2.2. Magnetic Resonance Elastography

The liver assessment included magnetic resonance imaging and elastography. All MR elastography was performed on a 1.5-T system (GE 450 1.5-T, Waukesha, WI). Images were analyzed on a GE Advantage workstation by drawing a freehand region of interest encompassing the entire measurable part of the liver.

## 3. Results

A total of 125 patients (56% male, 44% female) with Fontan circulation underwent surveillance with cardiac catheterization during the review period. Of these 125 patients, 53 (42%) were started on phosphodiesterase type 5 inhibitor therapy during the review period. Of these 53 patients, a total of 9 (17%) were placed on phosphodiesterase type 5 inhibitor therapy at the time of cardiac catheterization and later underwent posttherapy follow-up catheterization. Of the 53 patients, a total of 11 (21%) were placed on phosphodiesterase type 5 inhibitor therapy at the time of initial MR elastography testing with posttherapy follow-up MR elastography. The median time on phosphodiesterase type 5 inhibitor therapy for the two subgroups, catheterization and MR elastography evaluation, was 29.5 (4–60) and 26 (16–47) months, respectively. Primary diagnosis and dominant ventricle were variable.  Table 1 provides the summary of the patient demographics.

The mean Fontan pressure prior to phosphodiesterase type 5 inhibitor therapy was 15.4 mmHg and posttherapy was 13.3 mmHg, which was statistically significant (*p*=0.026), with an equivalent systemic systolic blood pressure. The mean wedge pressure prior to intervention was 9.2 mmHg and posttherapy was 8.6 mmHg (*p*=0.447). The mean transpulmonary gradient prior to phosphodiesterase type 5 inhibitor therapy was 6.2 mmHg and postphosphodiesterase type 5 inhibitor therapy was 4.8 mmHg (*p*=0.247). The mean systemic end-diastolic pressure (EDP) prior to phosphodiesterase type 5 inhibitor therapy was 7.8 mmHg and postphosphodiesterase type 5 inhibitor therapy was 7.2 mmHg (*p*=0.525). The mean indexed cardiac output prior to phosphodiesterase type 5 inhibitor therapy was 3.5 L/min/m^2^ and postphosphodiesterase type 5 inhibitor therapy was 3.3 L/min/m^2^ (*p*=0.394). The mean pulmonary vascular resistance prior to phosphodiesterase type 5 inhibitor therapy was 2.1 WU × m^2^ and postphosphodiesterase type 5 inhibitor therapy was 1.6 WU × m^2^ (*p*=0.321). The mean oxygen saturation in room air prior to phosphodiesterase type 5 inhibitor therapy was 91.6% and post-PDE5i therapy was 92% (*p*=0.672). The mean MR elastography stiffness prior to phosphodiesterase type 5 inhibitor therapy was 4.65 kPa and postphosphodiesterase type 5 inhibitor therapy was 4.63 kPa (*p*=0.958). Tables 2 and 3 provide the summary of these data. Three patients underwent pretherapy and posttherapy liver biopsies. Each liver biopsy was scored based on the modified Ishak congestive hepatic fibrosis (ICHF) scoring system [16], and the results are summarized in Table 4. Each patient had variable results, ranging from significant progression of their liver disease for one patient to improvement in their liver disease for another patient. There were no complications reported during any of the hemodynamic, MR elastography, or biopsy assessments.

## 4. Discussion

In this single-center study of patients with Fontan circulation who had been placed on pulmonary vasodilator therapy and subsequently underwent routine surveillance with cardiac catheterization or imaging of the liver by MR elastography, only Fontan pressure was statistically lower between the pretherapy and posttherapy groups. Although there was an improvement in wedge pressure, transpulmonary gradient, and end-diastolic pressure, these were not statistically significant. Several studies have been performed which profiled the use of pulmonary vasodilators and their immediate and short-term effects on the hemodynamics of patients with Fontan circulation. One study, by Mori et al., found statistical improvement in the pulmonary vascular resistance of patients following three months of oral outpatient sildenafil [13]. Another study, by Tunks et al., demonstrated an immediate improvement in pulmonary vascular resistance, Fontan pressure, and cardiac index by cardiac catheterization following administration of IV sildenafil [10]. Despite these findings, few studies have examined the long-term effect of oral pulmonary vasodilator therapy on the hemodynamic profiles of patients following Fontan operation. While the current study did not show a statistical improvement in many of these factors, this may be due to the low sample size used in our study. Our hemodynamic data do not suggest that routine use of phosphodiesterase type 5 inhibitor therapy is beneficial, even in patients with elevated Fontan pressures and low end-diastolic pressures. Potential therapeutic benefits may be demonstrated in future studies with large randomized controlled trials.

Liver fibrosis is a nearly universal finding during a routine biopsy in this patient population. In a study by Patel et al., 57 patients with Fontan circulation underwent biopsy; all but one was found to have hepatic fibrosis and 19 of these patients (33.3%) demonstrated bridging fibrosis or cirrhosis [16]. While liver biopsy has historically been the gold standard for measuring the degree of liver involvement for these patients, liver stiffness, as measured by MR elastography, has been shown to be correlated with histological fibrosis scoring systems and Fontan pressures [9, 15, 17]. Each of the patients who underwent a liver biopsy in our larger Fontan cohort was found to have some degree of liver fibrosis. Of the three individuals who underwent pretherapy and posttherapy liver biopsies to compare the effect of pulmonary vasodilators, the results were quite variable. One patient had significant progression of their liver disease, with severe portal fibrosis on their postbiopsy. In contrast, another patient demonstrated improvement, moving from 2A to 1 during the study period. This variability in findings may reflect the heterogeneity of liver disease within a patient's hepatic parenchyma, which has been well documented, and could suggest sampling errors with liver biopsy procurement [3, 4]. This study was one of the first to examine the effect of pulmonary vasodilator therapy on Fontan-associated liver disease using MRE of the liver and liver biopsies. Although we failed to demonstrate a statistical difference in liver stiffness for patients who were placed on pulmonary vasodilator therapy, there was individual improvement seen in some of our patients. Overall, these data do not demonstrate a clear benefit or harm of pulmonary vasodilator therapy on Fontan-associated liver disease, as characterized by liver stiffness and histological changes. To better delineate any effect of these drugs on Fontan-associated liver disease, larger prospective studies must be performed.

One of the major limitations of the current study was the small sample size, which affected our ability to achieve high power during our statistical analysis. Despite having a large sample of patients with Fontan circulation who had been started on pulmonary vasodilator therapy, only a few of these patients underwent posttherapy cardiac catheterization or MR elastography for an analysis to be performed. Another limitation of this study was the use of a retrospective analysis rather than a prospective study. By performing a retrospective analysis, we were unable to control any potential confounders, which ultimately likely led to poor statistical power. Another limitation was the variable time between pretherapy and posttherapy evaluations. Some of our patients remained on pulmonary vasodilator therapy for several years, while others were reevaluated just four months after initiation of pulmonary vasodilator therapy. Longer and more consistent therapy durations, which can be achieved easily in a prospective study, may demonstrate a stronger therapeutic effect.

The current study shows that the selective use of pulmonary vasodilators in patients with Fontan circulation is associated with a reduction in Fontan pressure. While not statistically significant, a reduction in transpulmonary gradient, end-diastolic pressure, and pulmonary vascular resistance was also seen. No improvement was seen in MR elastography liver stiffness scoring during our study. Some improvement, however, variable, was seen in the histological findings of the liver following this therapy. Overall, this study is unable to support the routine use of phosphodiesterase type 5 inhibitor therapy in Fontan patients, even in those with elevated Fontan pressures and low end-diastolic pressures. Ultimately, there is a strong need for larger randomized control trials to determine whether pulmonary vasodilator therapy is associated with improved long-term outcomes in patients with Fontan-associated liver disease.

## Figures and Tables

**Table 1:** Summary of patients who underwent pretherapy and posttherapy evaluations.

Characteristics	Cardiac catheterization evaluation (*n* = 9)	MR elastography evaluation (*n* = 11)
Male, *n* (%)	6 (66)	7 (64)
Age of patient (years)	17 (10–36)	19 (13–38)
Time since Fontan operation (months)	120 (28–318)	89 (45–316)
Time on PDE5i therapy (months)	29.5 (4–60)	26 (16–47)

Primary cardiac diagnosis: *n* (%)		
Hypoplastic left heart syndrome	3 (33)	3 (27)
Double outlet right ventricle	1 (11)	2 (18)
Double inlet left ventricle	2 (22)	1 (9)
Unbalanced AV canal	2 (22)	2 (18)
Tricuspid atresia	1 (11)	1 (9)
Pulmonary atresia with intact ventricular septum	0 (0)	1 (9)
Common atrium and ventricle	0 (0)	1 (9)

Systemic ventricle: *n* (%)		
Morphologically left ventricle	3 (33)	4 (36)
Morphologically right ventricle	6 (67)	6 (55)
Common ventricle	0 (0)	1 (9)

Data are provided as a median (range) or *n* (%).

**Table 2:** Hemodynamic data.

	Pretherapy	Posttherapy	*P* value
Fontan pressure (mmHg)	15.4 ± 3.3	13.3 ± 2.5	0.026
Wedge pressure (mmHg)	9.2 ± 2.6	8.6 ± 1.8	0.447
TPG (mmHg)	6.2 ± 1.9	4.8 ± 2.4	0.247
EDP (mmHg)	7.8 ± 2.3	7.2 ± 1.4	0.525
CI (L/min/m^2^)	3.5 ± 1.1	3.3 ± 1.0	0.394
PVR (wood units × m^2^)	2.1 ± 1.2	1.6 ± 0.6	0.321
Saturation (%)	91.6 ± 3.9	92.2 ± 2.2	0.672

Data are provided as a mean ± SD. TPG, transpulmonary gradient; EDP, end-diastolic pressure; CI, cardiac index; PVR, pulmonary vascular resistance.

**Table 3:** Magnetic resonance elastography data.

	Pretherapy	Posttherapy	*P* value
Liver stiffness (kPa)	4.65 ± 1.2	4.63 ± 1.7	0.958

Data are provided as a mean ± SD.

**Table 4:** Liver biopsy data.

	Patient 1	Patient 2	Patient 3
Pretherapy histological findings	Mild focal sinusoidal fibrosis, mild chronic inflammation, and mild sinusoidal dilation	Diffuse sinusoidal fibrosis, moderate portal fibrosis, mild chronic inflammation, and mild sinusoidal dilation	Moderately severe sinusoidal fibrosis, mild portal fibrosis, and mild sinusoidal dilation

Modified ISHAK (ICHF) score [16] pretherapy	1	3	2A

Posttherapy histological findings	Severe focal sinusoidal fibrosis, severe portal fibrosis, and mild sinusoidal dilation	Mild sinusoidal fibrosis mild portal fibrosis, moderate chronic inflammation	Mild sinusoidal fibrosis; moderate sinusoidal dilation

Modified ISHAK (ICHF) score [16] posttherapy	5	3	1

## Data Availability

The data used to support the findings of this study are included within the article.
